# Shifts in reclamation management strategies shape the role of exopolysaccharide and lipopolysaccharide‐producing bacteria during soil formation

**DOI:** 10.1111/1751-7915.13532

**Published:** 2020-01-09

**Authors:** Miljenka Vuko, Barbara Cania, Cordula Vogel, Susanne Kublik, Michael Schloter, Stefanie Schulz

**Affiliations:** ^1^ Research Unit Comparative Microbiome Analysis (COMI) Helmholtz Zentrum München Ingolstädter Landstr. 1 DE‐85764 Neuherberg Germany; ^2^ Chair of Soil Science Technical University of Munich Emil‐Ramann‐Straße 2 DE‐85354 Freising Germany; ^3^ Institute of Soil Science and Site Ecology Technical University of Dresden Pienner Str. 19 DE‐01737 Tharandt Germany

## Abstract

Polymeric substances produced by microbes play a key role for the development of soil aggregates. Here, we investigated the dynamics of bacterial families contributing to the formation of exopolysaccharides and lipopolysaccharides, major constituents of polymeric substances, at a managed land reclamation site of a post‐mining area. We collected soil samples from the initial and the agricultural management phase and expected a peak in the abundance of bacteria capable for exopolysaccharide and lipopolysaccharide production at the points of the biggest disturbances. We used shotgun metagenomic sequencing in combination with measurements of exopolysaccharide concentrations. Our results underline the importance of exopolysaccharide and lipopolysaccharide‐producing bacteria after nutrient input combined with structural disturbance events, caused here by the initial planting of alfalfa and the introduction of a tillage regime together with organic fertilization in the agricultural management phase. Moreover, the changes in management caused a shift in the exopolysaccharide/lipopolysaccharide‐producing community. The initial phase was dominated by typical colonizers of oligotrophic environments, specifically nitrogen fixers (*Rhizobiaceae*, *Comamonadaceae*, *Hyphomicrobiaceae*), while bacteria common in agricultural soils, such as *Sphingomonadaceae*, *Oxalobacteraceae* and *Nitrospiraceae,* prevailed in the agricultural management phase.

## Graphical abstract

We investigated the dynamics of bacterial families contributing to the formation of exopolysaccharides and lipopolysaccharides at a managed land reclamation site of a post‐mining area. Our results underline the importance of exopolysaccharide and lipopolysaccharide‐producing bacteria after nutrient input combined with structural disturbance events, caused here by the initial planting of alfalfa and the introduction of a tillage regime together with organic fertilization in the agricultural management phase.

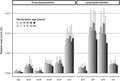

## Introduction

Soil development is strongly influenced by the interaction of soil biota with the mineral phase of the parent material (Banfield *et al.*, 1999; van Breemen *et al.*, 2000; Schaaf *et al.*, 2011). This interaction results in the formation of aggregates, the basic units of soil structure (Lynch and Bragg, 1985).

During soil development, extracellular polymeric substances (EPS) excreted by microorganisms play an important role (Costa *et al.*, 2018). These substances trigger the initial colonization of abiotic surfaces by planktonic cells and thus enable the formation of complex network structures such as biofilms and soil crusts (Velmourougane *et al.*, 2017). Furthermore, EPS are able to entrap nutrients and protect microbes against environmental stresses (Flemming, 2016; Flemming *et al.*, 2016; Costa *et al.*, 2018) by generating favourable niches (Velmourougane *et al.*, 2017). Besides the key role that EPS play in the formation of biofilms and soil crusts, these substances also entrap soil particles (Costerton *et al.*, 1995). Thus, they are assumed to contribute to overall soil aggregation and structure stabilization at the initial stage of soil development.

Major fractions that determine adhesive properties of EPS are exopolysaccharides and lipopolysaccharides (Velmourougane *et al.*, 2017). Exopolysaccharides secreted by microbes are high‐molecular‐weight sugars with great variation in structure and function (Sutherland, 2001). Due to their high carbon content, the production is energetically expensive and relies on a sufficient supply of carbon. In general, the biosynthesis and export of exopolysaccharides follows one of three pathways: (i) Wzx and Wzy‐dependent, (ii) ATP‐binding cassette (ABC) transporter‐dependent and (iii) synthase‐dependent pathway (Whitney and Howell, 2013). In the Wzx/Wzy‐dependent pathway, oligosaccharides are polymerized and translocated through the inner plasma membrane by the integral inner membrane proteins Wzx, which acts as a flippase, and Wzy, which acts as a polymerase. Oligosaccharides are subsequently exported through the outer membrane by a protein complex formed by Wzc and Wza (Pereira *et al.*, 2015). In contrast, polysaccharides assembled by ABC transporters are polymerized inside the cell and exported by two different protein domains: a transmembrane and a nucleotide‐binding domain (Cuthbertson *et al.*, 2010), while in the synthase‐dependent pathway, the polysaccharide is simultaneously polymerized and exported across the cell membrane (Pereira *et al.*, 2015).

Lipopolysaccharides are complex glycolipids attached to the outer membrane of most Gram‐negative bacterial cells (Jacques, 1996). Lipopolysaccharides are composed of the lipid A, a core oligosaccharide and a long‐chain O‐antigenic polysaccharide, and they provide cell integrity, and, in many cases, interact with surfaces in proximity (Esteban *et al.*, 2014; Whitfield and Trent, 2014). Components of lipopolysaccharides are synthesized in the cytoplasm, assembled in the periplasm and exported across the cell membrane by a complex lipopolysaccharide protein machinery (Wang and Quinn, 2010).

Despite their importance in the initial colonization of parental materials and subsequent soil formation, there is still a lack of knowledge on which microbes drive the formation of extracellular polymeric substances, and which are the important pathways for exopolysaccharide and lipopolysaccharide expression at different stages of soil development. Thus, the aim of this study was to identify important microbial producers for exopolysaccharides and lipopolysaccharides during soil formation. We made use of a post‐mining reclamation area where a chronosequence of soils in different development stages was present as a result of continuous open‐cast mining and subsequent reclamation. In this chronosequence, the soils originated from the same substrate and were managed the same way, but differed in the time since they were formed, which is the basic precondition of a chronosequence ‘space‐for‐time’ substitution approach (Walker *et al.*, 2010).

Soils reclaimed after a drastic disturbance event such as open‐cast mining are low in nutrients and microbial diversity, and have poor physiochemical properties (Shrestha *et al.*, 2009). Therefore, microbes, as ‘architects’ of soil on the micro level, play a crucial role in soil development of reclaimed areas (Šourková *et al.*, 2005; Totsche *et al.*, 2010). Thus, we have to understand their dynamics during the reclamation to ensure proper functioning of the reclaimed soil.

The aim of the reclamation used in this study was to generate soils that could be used for intensive farming. Thus, the management during the reclamation included the mixing of parental material with soil used for agriculture, followed by the cultivation of alfalfa (*Medicago sativa*) for three years to improve nitrogen and carbon stocks in the soils, as well as to promote soil structuring (Boldt‐Burisch *et al.*, 2015). The three‐year initial stage was followed by a crop rotation, which was accompanied with intensive tillage and fertilization. We followed bacterial exopolysaccharide and lipopolysaccharide producers in a chronosequence covering a period of 24 years and focused on six sites in different developmental stages during this time. We measured the concentration of exopolysaccharides in the bulk soil of the different age classes selected and performed metagenomic sequencing with subsequent bioinformatic analysis focusing on genes involved in both the synthesis and export of exopolysaccharides (e.g. alginate, colanic acid, levan) and lipopolysaccharides.

As (i) bacteria produce extracellular polymeric substances mostly to protect themselves from environmental stresses, and (ii) high loads of accessible carbon and nutrients are necessary for the production of these compounds, we speculated that there would be a peak in the abundance of bacteria capable of exopolysaccharide and lipopolysaccharide production at the time point of the biggest disturbance, specifically when the alfalfa was first planted and when the alfalfa system was changed to a cropping system associated with tillage and high input of organic fertilizers.

## Results

### Exopolysaccharide content

**Figure 1:**
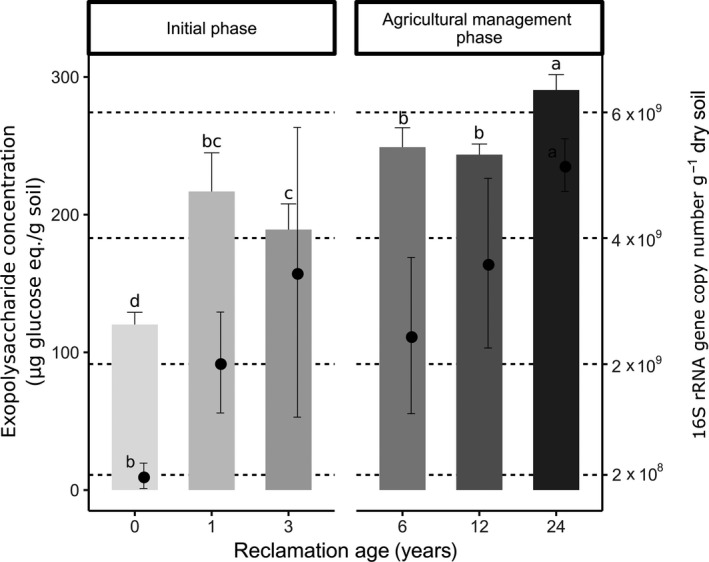
Concentration of exopolysaccharides and bacterial abundance in the two reclamation phases. Exopolysaccharide contents are shown as bars with values presented on the left y‐axis, while the abundance of bacteria is depicted as points with values presented on the right y‐axis. Bars and points represent the means of triplicates, and error bars represent standard deviations. The concentrations of exopolysaccharides were calculated as equivalents of glucose used as the standard, per gram of dry soil. Bacterial abundances were calculated as 16S rRNA gene copy numbers per gram of dry soil. Letters above bars and points represent pairwise comparisons adjusted with the Benjamini–Hochberg correction showing the significant differences between reclamation ages after a robust ANOVA test with trimmed means (*P *< 0.05).

The concentration of exopolysaccharides was significantly higher in soil samples from sites after the beginning of the reclamation compared to soil samples from the initial stage at reclamation age zero years (RA0) (Fig. 1). During the initial phase, concentration increased almost twofold from RA0 (120.21 ± 8.9 µg g^−1^ dw soil) to reclamation age one year (RA1) (216.9 ± 28.1 µg g^−1^ dw soil). A slight decrease was observed at RA3 (189.18 ± 18.7 µg g^−1^ dw soil). During the agricultural management phase of the reclamation, only slight increases were visible. Concentration at RA6 increased to 249 ± 14.1 µg g^−1^ dw soil, and the highest concentrations were observed at RA24 (290.54 ± 11.2 µg g^−1^ dw soil). Measurements of the proteinaceous components of extracellular polymeric substances showed a similar trend (Fig. S2): the concentration doubled during the first year of reclamation and was almost three times higher after 24 years of reclamation.

### Bacterial abundance

Abundance of bacteria was calculated as 16S rRNA gene copy numbers per gram of dw soil (Fig. 1) by means of qPCR. Bacterial abundance was low in the beginning of the reclamation (2.26 × 10^8^ ± 2 × 10^8^ copies g^−1^ dw soil at RA0) and steadily increased during the initial phase to 3.46 × 10^9^ ± 2.3 × 10^9^ copies g^−1^ dw soil at RA3. Following the transition to the agricultural management phase, abundance dropped to 2.46 × 10^9^ ± 1.2 × 10^9^ copies g^−1^ dw soil at RA6. The highest abundance of 16S rRNA genes was obtained from RA24 (5.16 × 10^9^ ± 4.18 × 10^8^ copies g^−1^ dw soil).

### Shotgun sequencing summary

The total number of filtered sequencing reads obtained from 18 libraries was 45.8 million. The number of reads obtained per sample ranged from 1.6 to 3.7 million, with read length ranging from 292.23 to 296.96 bp. For the non‐template control, 212 reads were obtained (and excluded from further analysis), indicating very low contamination during DNA extraction and metagenomic library preparation (Fig. S3). The coverage of metagenomes based on the read redundancy values (as calculated by the Nonpareil algorithm) ranged between 0.8 and 5% (Table S2). As expected, the highest coverage was obtained for samples from RA0, indicating the lowest diversity in those samples. The highest diversity was measured in samples from RA1, and it was significantly higher compared to sites with longer reclamation periods.

Taxonomic annotation was accomplished by blasting all filtered reads against the NCBI‐NR database, which resulted in 69 to 79% of reads annotated at the superkingdom level. Most reads corresponded to *Bacteria* (63 to 75%), 2.8 to 3.9% of the reads could be assigned to *Eukaryota*, and less than 1% to *Archaea* and *Viruses*. In total, 384 bacterial families were detected, while reads assigned to genes involved in exopolysaccharide and lipopolysaccharide formation were found in 245 bacterial families. For the most abundant families, reads were largely assigned to one to three genera (Fig. S4). To confirm the data from the taxonomic assignment of all reads, we compared it with the assignment of the 16S rRNA gene using Ribotagger (Xie *et al.*, 2016) and SILVA database. As expected, the relative abundance of reads assigned to the 16S rRNA gene was less than 0.5% of all filtered reads and most of them were not assigned to family level. However, the phylogenetic analysis of reads assigned to the 16S rRNA gene confirmed most of the dominant bacterial groups obtained by the analysis of all assigned reads (Fig. S5).

### Relative and total abundances of genes involved in exopolysaccharide and lipopolysaccharide synthesis and excretion

As most annotated reads were assigned to *Bacteria*, the analysis of exopolysaccharide and lipopolysaccharide genes was performed for bacterial reads only. To target genes specific for exopolysaccharide and lipopolysaccharide (Table S1) production and excretion, we used a combination of hidden Markov models (HMMs), which were based on data from the online Kyoto Encyclopedia of Genes and Genomes (KEGG) database. As a consequence of their low abundance, all reads assigned to *algE*, *epsG*, *epsA* and *wcaK/amsJ* (less than 10 reads per sample) were excluded from further analysis.

**Figure 2:**
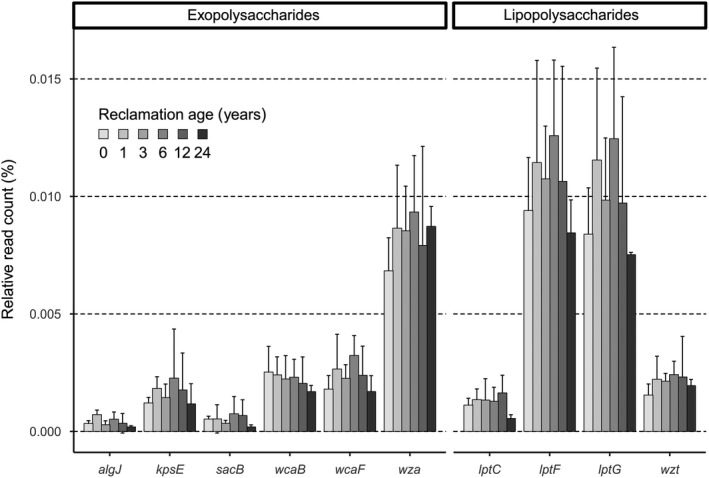
The relative read count of genes involved in exopolysaccharide and lipopolysaccharide biosynthesis. Bars represent the means of triplicates per reclamation age with a standard deviation. Differences between reclamation ages were tested using the robust ANOVA with trimmed means, which showed no significant difference (*P* > 0.05).

The total relative abundance of all exopolysaccharide and lipopolysaccharide genes analysed here ranged from 0.032 to 0.047% of all filtered bacterial reads per sample. Generally, reads that could be assigned to genes involved in lipopolysaccharide formation were higher in relative abundance mainly at the early stages of reclamation, compared to the reads that were assigned to genes driving exopolysaccharide production. The most abundant genes were *lptF* and *lptG,* which encode for parts of the permease that exports lipopolysaccharides, followed by *wza*, a gene encoding for the polysaccharide export outer membrane protein (Fig. 2). Even though the reclamation age did not significantly influence relative gene abundances, seven out of ten analysed genes showed the same trend. These genes include *kpsE*, *wcaF*, *wza*, *lptF*, *lptG* and *wzt*, which showed a slight increase in soils from RA1 compared with RA0, and a peak following the transition to the agricultural management phase at RA6. At RA6, these genes comprised 0.0023, 0.0032, 0.0093, 0.0126, 0.0125 and 0.0024% of mean relative abundance respectively. The *sacB* gene also had a peak at RA6, where it comprised 0.0008% of mean relative abundance. Relative abundances of all genes, with the exception of *wza*, were lowest in soil samples obtained from RA24.

Additionally, we roughly estimated absolute abundances of exopolysaccharide and lipopolysaccharide genes by comparing them to the 16S rRNA absolute abundances measured by qPCR. The most abundant genes, namely *wza*, *lptF*, *lptG*, *wzt*, *wcaB* and *wcaF,* showed a similar trend as 16S rRNA gene copy numbers: the strongest increase in soils from RA1, a slight decrease in RA6 and increases in RA12, especially RA24 (Fig. S6).

### Taxonomy of potential exopolysaccharide and lipopolysaccharide producers

**Figure 3:**
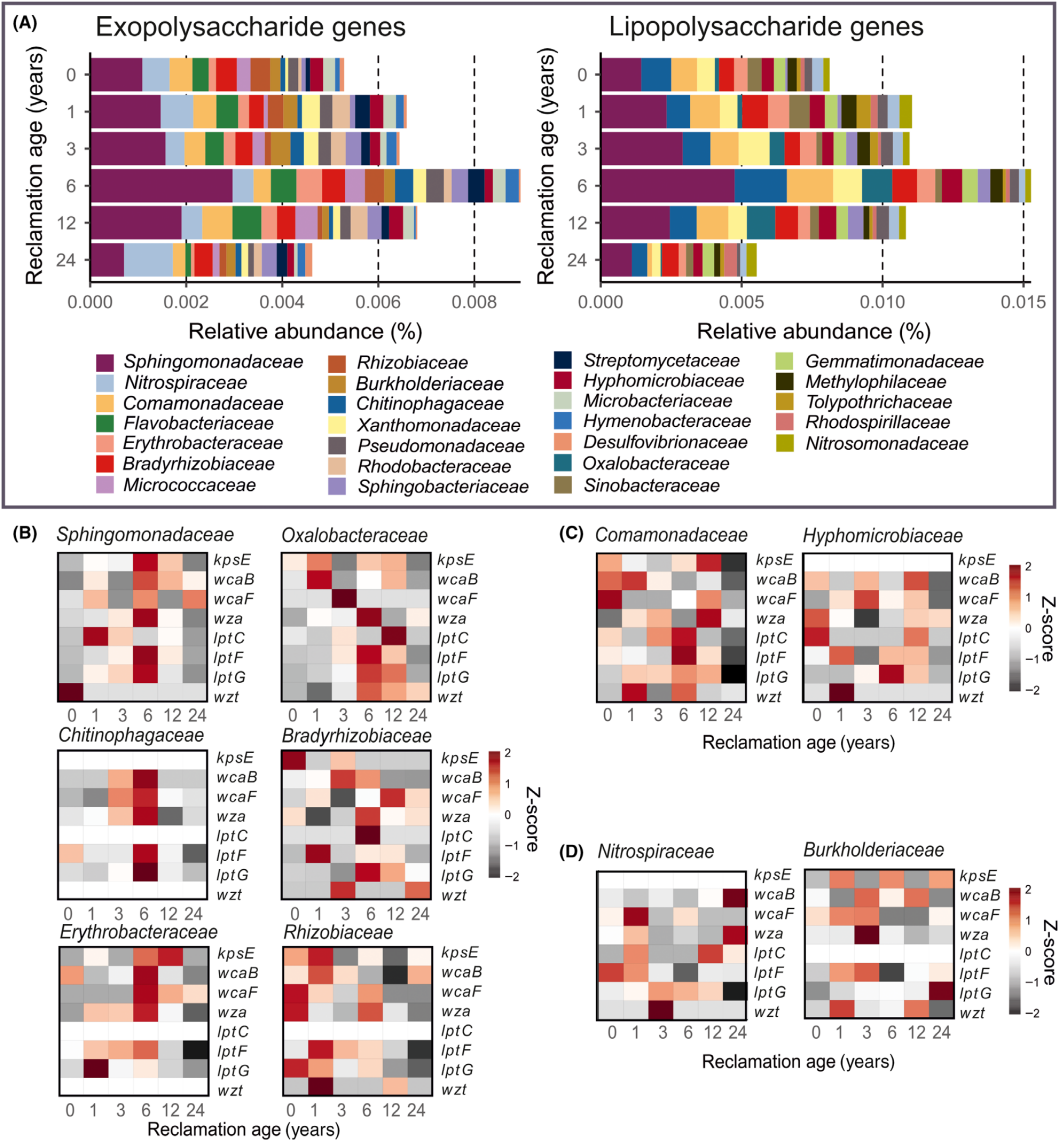
Taxonomic affiliation of analysed exopolysaccharide and lipopolysaccharide genes – distribution of gene copies among 10 bacterial families harbouring the most genes per reclamation age. **A.** Mean relative abundances of exopolysaccharide and lipopolysaccharide genes summed up and divided by the number of genes: six and four for exopolysaccharide and lipopolysaccharide genes respectively. **B, C, D.** Heatmap representations of relative gene abundances harboured by particular bacterial families based on data normalized by Z score transformation. In each family, genes are represented in rows, and the amount of gene per sample is expressed as a unit of standard deviation from the mean of gene abundance in all samples, which is normalized to zero (white). Z score is positive (red) when the sample is above mean, and negative when the sample is below mean (grey). Shown are eight genes with the highest abundance: *kpsE, wcaB, wcaF, wza, lptC, lptF, lptG* and *wzt*, encoding for the capsular polysaccharide export permease, colanic acid biosynthesis, polysaccharide export outer membrane protein, lipopolysaccharide export permease and inner membrane protein, and lipopolysaccharide transport system ATP‐binding protein, respectively. **B.** Families with most exopolysaccharide/lipopolysaccharide genes assigned in reclamation ages 1 and/or 6 **C.** Families with a decrease of exopolysaccharide, but an increase of lipopolysaccharide genes assigned in reclamation age 6 **D.** Families with a decrease of exopolysaccharide/lipopolysaccharide genes assigned in reclamation age 6.

Overall, 245 different families were involved in the formation of exopolysaccharides and lipopolysaccharides. Further analysis focused on the most abundant carriers of genes involved in exopolysaccharide and lipopolysaccharide production in all reclamation ages. We filtered 10 bacterial families harbouring the most genes per each reclamation age and found 26 that were phylogenetically affiliated with exopolysaccharide and lipopolysaccharide genes (Fig. 3a). Most of these families were involved in both processes (16 families). Exclusive exopolysaccharide producers involved *Flavobacteraceae*, *Micrococcaceae*, *Rhodobacteraceae*, *Desulfovibrionaceae*, *Microbacteriaceae*, *Streptomycetaceae* and *Hymenobacteraceae*, whereas *Gemmatimonadaceae*, *Tolypothrichaceae*, *Sinobacteraceae*, *Rhodospirillaceae*, *Nitrosomonadaceae* and *Methylophilaceae* were involved only in lipopolysaccharide production.

Of the families capable to produce both compound classes, *Sphingomonadaceae* were dominating at all RAs. The only exception were exopolysaccharide producers at RA24. Here, *Nitrospiraceae* were more abundant compared to *Sphingomonadaceae.* However, *Nitrospiraceae,* together with *Comamonadaceae,* had an important role as a source of exopolysaccharides in other RAs too, as they were among the top three most abundant potential exopolysaccharide producers. In contrast, the second most abundant family for lipopolysaccharide production was *Chitinophagaceae* at RA0, RA6 and RA24, *Comamonadaceae* at RA1 and RA12, and *Xanthomonadaceae* at RA3. *Nitrospiraceae* played only a minor role in lipopolysaccharide production. When comparing other, less abundant families involved in both exopolysaccharide and lipopolysaccharide production, we also observed differences in their relative abundances between sites of different reclamation ages.

A detailed analysis of the different genes harboured by the major groups involved in exopolysaccharide and lipopolysaccharide production revealed a clear shift in population structure over time (Fig. 3B). For most bacterial families, the highest relative abundance of genes driving exopolysaccharide and lipopolysaccharide production was found at RA6, including *Sphingomonadaceae*, *Oxalobacteraceae*, *Chitinophagaceae*, *Bradyrhizobiaceae* and *Erythrobacteraceae.* For *Sphingomonadaceae,* this was most evident for the capsular polysaccharide export permease‐encoding *kpsE* and the polysaccharide biosynthesis and export protein encoding *wza*. Among the analysed lipopolysaccharide genes, the same trend for *Sphingomonadaceae* was observed with *lptF* and *lptG.* A similar trend was observed for the other four families. However, *Chitinophagaceae* were lacking *wzt* and *kpsE* genes, while the highest relative abundance of reads for *kpsE* was found for *Erythrobacteraceae* at RA12. The importance of the cultivation of alfalfa is evident in *Rhizobiaceae,* as for this family, seven genes (*kpsE*, *wcaB*, *wcaF*, *wza*, *lptC*, *lptG* and *wzt*) were most abundant in RA0 and RA1.

*Comamonadaceae* had an increase of relative abundance of lipopolysaccharide genes, but a decrease of exopolysaccharide genes following the transition to the second reclamation phase at RA6 (Fig. 3C). This family showed a high potential to produce exopolysaccharides in the beginning of the reclamation, and a potential to produce lipopolysaccharides (namely LptBFGC export permease and lipopolysaccharide transport system ATP‐binding protein) at RA6. For *Hyphomicrobiaceae,* a high relative abundance at RA6 was only found for *lptF* and *lptG* genes, but not for *lptC* and *wzt*. Interestingly, for both families we observed a strong decrease of relative abundance values for genes driving exopolysaccharide and lipopolysaccharide formation in a later stage of the reclamation at RA24.

For some families, including *Nitrospiraceae* and *Burkholderiaceae,* a decrease in relative abundance was observed for genes triggering both exopolysaccharide and lipopolysaccharide production at RA6 (Fig. 3D). For *Nitrospiraceae,* the relative abundance of *wza* and *wcaB* genes, encoding for polysaccharide and colanic acid biosynthesis, respectively, peaked at RA24. For *Burkholderiaceae,* most reads for these genes, including *wza*, *wcaF, wcaB* and *lptF* genes*,* were assigned to in RA3.

### Comparison of exopolysaccharide/lipopolysaccharide producers with the overall relative abundance of the different families

**Figure 4:**
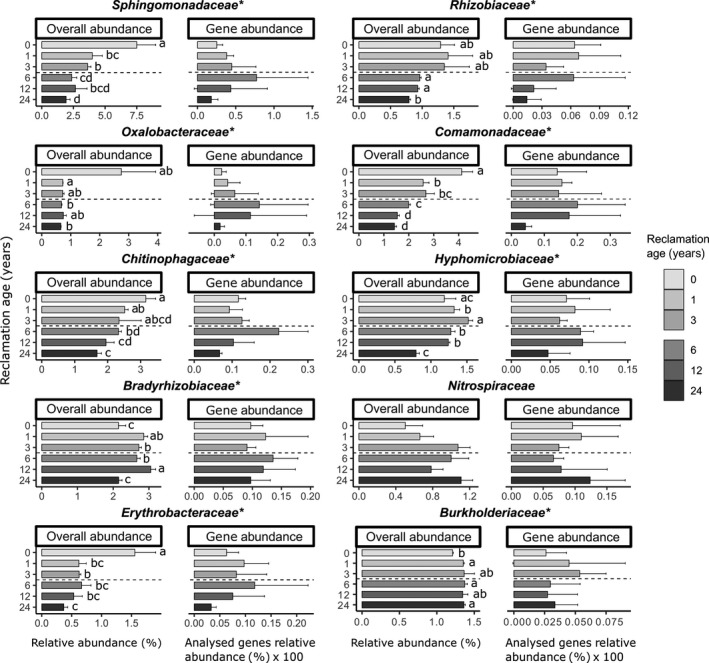
Comparison of relative abundances of particular highly abundant bacterial families with their potential to produce exopolysaccharides and lipopolysaccharides. Bars represent the means of triplicates per reclamation age with a standard deviation. Marked families (*) were found to be significantly influenced by reclamation age in their relative abundance (Robust ANOVA with trimmed means, *P* < 0.05), and letters next to the bars represent pairwise comparisons adjusted with the Benjamini–Hochberg correction. Relative abundances of exopolysaccharide and lipopolysaccharide genes are shown as percentages multiplied by 100.

A comparison of bacterial families carrying exopolysaccharide and/or lipopolysaccharide genes with their overall relative abundance in samples from all reclamation ages is presented in Fig. 4. The amount of exopolysaccharide and lipopolysaccharide genes assigned to *Sphingomonadaceae, Comamonadaceae, Chitinophagaceae, Oxalobacteraceae* and *Erythrobacteraceae* peaked at RA6. However, overall relative abundances of these families were steadily decreasing during the reclamation process.

For *Sphingomonadaceae,* the highest relative abundance of 7.3% was found at RA0. In samples from RA24, the abundance of *Sphingomonadaceae* decreased by a factor of four, (1.92% of all reads assigned to Bacteria). Relative abundance of *Comamonadaceae* and *Chitinophagaceae* families was also continuously decreasing from 4.1 to 1.4%, and 3.2 to 1.7%, respectively, at RA0 and RA24, while *Oxalobacteraceae* and *Erythrobacteraceae* sharply decreased already at RA1. For *Bradyrhizobiaceae,* the highest relative abundance was measured at RA12, but most exopolysaccharide/lipopolysaccharide genes assigned to this family were observed in samples from RA1 and RA6. Similar to *Bradyrhizobiaceae*, most exopolysaccharide/lipopolysaccharide genes assigned to *Rhizobiaceae* were observed in reclamation ages RA1 and RA6, while the overall abundance of this family decreased in the agricultural management phase compared to the initial phase. The abundance of *Burkholderiaceae* increased after the beginning of the initial phase, but exopolysaccharide/lipopolysaccharide genes assigned to this family decreased after the transition to the agricultural management phase. *Nitrospiraceae* was the only family among the highly abundant ones that increased both in the overall abundance and in the amount of assigned exopolysaccharide/lipopolysaccharide genes at RA24.

## Discussion

In this study, we followed a succession of potential exopolysaccharide and lipopolysaccharide‐producing bacteria during the reclamation of a post‐mining site, where soil formation was accelerated by specific forms of management. In the initial phase of the reclamation, a legume (*Medicago sativa*) was grown for three years. In this phase, we observed an increase in the concentration of exopolysaccharides, bacterial abundance and bacterial potential to produce exopolysaccharides and lipopolysaccharides. In the following agricultural management phase, the soil was tilled and fertilized, and cultivated with a crop rotation system. After the transition to the second phase, the abundance of bacteria decreased at RA6, whereas the exopolysaccharide concentration and relative abundances of genes driving exopolysaccharide and lipopolysaccharide formation increased. In fact, the relative abundance of seven out of ten analysed genes reached the highest values at RA6. After 24 years of reclamation, exopolysaccharide concentration and the abundance of bacteria reached the highest values, whereas the relative abundance of genes involved in the biosynthesis of exopolysaccharides and lipopolysaccharides decreased.

### Setting the stage for soil formation in the initial reclamation phase

Biological activation of soil in the initial phase is following the cultivation of alfalfa and is reflected in the increasing exopolysaccharide concentrations, and bacterial, as well as fungal abundance, as was shown in a previous study on the same reclamation site (Pihlap *et al.*, 2019). Prior to the alfalfa growth (RA0), exopolysaccharide concentrations were comparable to other sites with scarce vegetation, such as bare fallow land (Redmile‐Gordon *et al.*, 2014). After one year of alfalfa cultivation, we observed an almost twofold increase of exopolysaccharide concentration (Fig. 1), which is, however, still significantly lower than, for example grassland soils (Redmile‐Gordon *et al.*, 2014). The latter values were also not reached after 3 years of alfalfa cultivation.

Similar to exopolysaccharide concentrations, relatively low bacterial abundance prior to alfalfa cultivation is not surprising, as similar results were observed in different initial ecosystems (Esperschütz *et al.*, 2011; Dini‐Andreote *et al.*, 2014). It is difficult to assess whether the increase in bacterial biomass in RA1 can be directly linked to the increased exopolysaccharide concentrations, as the various sugar sources in soil make it difficult to determine their origin (e.g. fungi and plants can also produce exopolysaccharides) (Gunina and Kuzyakov, 2015). However, we also observed an increase in the relative abundance of bacterial exopolysaccharide and lipopolysaccharide genes at RA1, and as our sampling strategy excluded the sampling of plant root materials, it suggests that bacteria play an important role in the formation of exopolysaccharides at least at the early stages of reclamation. Additionally, in a recent meta‐analysis including 64 studies, it was concluded that only 20% of all sugars in soil originate from a primary source (decomposition of plant litter and rhizodeposits), while 80% originate from secondary sources (microorganisms and their residues) (Gunina and Kuzyakov, 2015). Unlike exopolysaccharides, commonly used extraction methods for lipopolysaccharides are based on biological assays developed for detecting lipopolysaccharides in human or animal tissues, or from pure cell cultures (Stromberg *et al.*, 2017), and are not well adapted for extraction from environmental samples (Tunlid and White, 1992; Rylander, 2002). Consequently, lipopolysaccharide extractions and measurements were not included in this study.

As a result of alfalfa cultivation, we observed an expected strong overall increase of *Rhizobiaceae,* particularly *Sinorhizobium*, at RA1. The highest relative abundance values for genes involved in exopolysaccharide and lipopolysaccharide formation for *Rhizobiaceae* were found in the initial development stage, mainly for *kpsE*, *wcaB*, *lptF* and *wzt*, indicating the importance of this group of bacteria for soil development, for example soil structure formation, alongside its abilities to improve nitrogen and phosphorus content in soil. A similar result was observed with *Hyphomicrobiaceae,* which also belong to *Rhizobiales*. Biofilm formation is an important survival strategy for rhizobia, allowing them to live not only on plant surfaces, but also on soil mineral surfaces under different environmental conditions (Russo *et al.*, 2015). Since lipopolysaccharides determine cell adhesiveness and cell–cell aggregation, they also play a central role in biofilm formation and were found to be the regulation factor in biofilm formation of *Rhizobium leguminosarum* (Russo *et al.*, 2015). Many species belonging to the *Hyphomicrobiaceae* are known to thrive only in the presence of low concentrations of suitable carbon (Oren and Xu, 2014), which could enable them to live in low‐nutrient initial ecosystems.

Overall, the highest increase of relative exopolysaccharide and lipopolysaccharide gene abundances at RA1 was observed for the most abundant genes – *wza*, *lptF* and *lptG*; a trend also seen when considering the total bacterial abundance (Fig. S6). The *wza* gene encodes for the ring‐like outer membrane protein Wza, which, together with polysaccharide co‐polymerase protein families (Cuthbertson *et al.*, 2009), forms a protein channel through which polysaccharide chains are exported out of the cell (Collins *et al.*, 2007; Whitney and Howell, 2013). A relatively high abundance of this gene is not surprising, since Wza is a part of the Wzx/Wzy‐dependent pathway, which is the most widely distributed polysaccharide transport mechanism present in a large number of bacteria (Whitfield, 2010). Similarly, *lptF* and *lptG* are genes encoding for two essential inner membrane proteins in Gram‐negative bacteria, which transport lipopolysaccharides to the outer cell membrane (Sperandeo *et al.*, 2008). These proteins are a part of the Lpt protein machinery that functions as an ABC transporter to export lipopolysaccharides outside of the cell, and are consequently widely distributed among Gram‐negative bacteria. In contrast, the *lptC* gene codes for another protein of the Lpt machinery that is not found in all bacteria; therefore, its lower abundance is not surprising (Haarmann *et al.*, 2010; Tran *et al.*, 2010; Whitfield and Trent, 2014).

An example of Gram‐negative bacteria are *Sphingomonadaceae*, the most abundant family in the initial reclamation phase (Fig. 4), and the most prominent carrier for exopolysaccharide and lipopolysaccharide genes (Figs 2 and 3). This family includes genera, particularly *Sphingobacterium*, which have been proven to have a great ability to stabilize soil and increase aggregate strength, especially in the rhizosphere of alfalfa (Caesar‐TonThat *et al.*, 2007). In addition, *Sphingomonadaceae* have characteristic sphingolipids with strong adhesive properties in the outer membrane, and *Sphingomonas*, the type genus, which was also the dominant genus found within *Sphingomonadaceae* in all RAs, is known for the production of exopolysaccharides named sphingans (Caesar‐TonThat *et al.*, 2007; Glaeser and Kämpfer, 2014). Another family found in high abundance in the beginning of the reclamation is *Comamonadaceae* (particularly *Variovorax* genus), which is a large and phenotypically diverse bacterial family commonly found in soils, containing some photoautotrophic genera as well (Willems, 2014). Furthermore, *Comamonadaceae* have been found in the early stages of biofilm development and are suggested to play a role in the initial cell adhesion and colonization processes (Di Gregorio *et al.*, 2017).

### Transition to the agricultural management phase – survivors with a higher potential for exopolysaccharide and lipopolysaccharide production

Transition to the agricultural management phase (RA6) went along with several changes in the soil ecosystem: a decrease of bacterial abundance, an increase in relative abundances of exopolysaccharide and lipopolysaccharide genes, and a slight increase of the exopolysaccharide concentration. The start of tillage practices in this phase most likely disturbed niches shaped by microbes during early soil development and disrupted aggregates into mineral particles and organic residue, increasing the available surface area for microbial colonization, and diluting accumulated nutrient pools by mixing with nutrient‐low subsoil (Johnson and Hoyt, 1999; Balota *et al.*, 2003; Costa *et al.*, 2018). At the same time, the organic fertilizer added after the transition increased C availability, hence boosting the microbial community, and fuelling exopolysaccharide and lipopolysaccharide producers, since the production of these substances requires high amounts of C and energy (Costa *et al.*, 2018).

Interestingly, Pihlap *et al. *(2019) did not observe significant differences in aggregate sizes in the sites of the same chronosequence due to the cementing effect of high carbonate contents. However, seven out of ten genes that have been analysed here had highest relative abundances at RA6, and all of them, with the exception of *wza*, declined during later reclamation stages (Fig. 2). Furthermore, authors of the previous study observed a large increase of dissolved organic carbon and nitrogen, as well as organic carbon stored within the smallest aggregates at RA6, which are likely to be the aggregate fraction mostly affected by bacterial exopolysaccharide and lipopolysaccharide excretion, and biofilm formation (Six *et al.*, 2004). This formation of biofilm extracellular polymeric substances as a strategy of protection from environmental stressors is well known for microbes living in disturbed ecosystems (Rossi and De Philippis, 2015), such as the one here created by deep tillage. By excreting these substances, bacterial cells can surround themselves with clay particles (Burns and Davies, 1986), making their adhesive properties beneficial for soil structural properties via gluing soil particles together (Costa *et al.*, 2018). At RA6, we observed an increase in the relative abundances of genes involved in exopolysaccharide biosynthesis mostly for two families belonging to the order *Sphingomonadales*: *Sphingomonadaceae* and *Erythrobacteraceae* (Tonon *et al.*, 2014), indicating a change in the exopolysaccharide/lipopolysaccharide‐harbouring bacteria compared to initial stages of reclamation dominated by, for example *Rhizobiaceae* and *Comamonadaceae*. Although the total bacterial abundance was lower at RA6 compared with RA24, the relative increase in exopolysaccharide/lipopolysaccharide‐producing families in the community, together with the addition of C and therefore energy, resulted in an accelerated production of exopolysaccharides. This corroborates our assumption that the change in management alters the microbial community towards one that is more efficient in producing exopolysaccharides and lipopolysaccharides.

### Stable environmental conditions – a lower need for exopolysaccharide and lipopolysaccharide production

The decrease in the relative abundance of genes involved in exopolysaccharide and lipopolysaccharide biosynthesis at RA3 and RA24 could indicate that bacterial communities adapt to the changes in the environment.

During the initial reclamation phase with alfalfa cultivation, the relative abundances of rhizobia, namely *Rhizobiaceae, Bradyrhizobiaceae* and *Hyphomicrobiaceae*, are increasing at RA1 and RA3. Yet, we observed a decrease in the relative abundance of genes involved in exopolysaccharide and lipopolysaccharide production for these families at the same stage (Fig. 4). As it is known that exopolysaccharides and lipopolysaccharides play an important role in the initial establishment of successful rhizobia‐legume symbiosis (Abdian and Zorreguieta, 2016), the increased relative abundance of exopolysaccharide and lipopolysaccharide genes in RA1 is not surprising. At RA24, we observed a high overall abundance, as well as increased relative exopolysaccharide and lipopolysaccharide gene abundances for the *Nitrospiraceae* family, mainly the *Nitrospira* genus. Due to the ability of this family to perform an important step in nitrogen cycling, its members are readily found in agricultural soils (Xia *et al.*, 2011), and many of them are known to produce large amounts of exopolysaccharides (Daims, 2014). With that, *Nitrospiraceae* replaced *Sphingomonadaceae* as the most abundant exopolysaccharide producer at RA24, which underlines the advantage of bacteria involved in important nutrient cycles and exopolysaccharide/lipopolysaccharide production over solely exopolysaccharide/lipopolysaccharide producers in agricultural soils.

The total bacterial abundance measured by qPCR increased during the initial and agricultural management phase and was highest at RA24 (Fig. 1), which was also reflected in the estimated absolute abundances of the most abundant exopolysaccharide/lipopolysaccharide genes (Fig. S6). However, the relative abundances of all analysed bacterial exopolysaccharide and lipopolysaccharide genes, except *wza*, decreased at the same site. This result indicates that the bacterial community grows in abundance, and eventually adapts and stabilizes with agricultural practices. This is in accordance with other studies showing a stable community under long‐term treatments (at least on the DNA level) (de Vries *et al.*, 2015; Cania *et al.*, 2019). Moreover, soil organic carbon concentrations peaked at RA6 and decreased at RA12 and RA24 (Pihlap *et al.*, 2019), likely because organic fertilizers were applied only in the 4th and 7th year of the reclamation process. Therefore, the peak in exopolysaccharide concentration at RA24 underlines the importance of soil bacteria, through exopolysaccharide accumulation, for the improvement of soil quality and structure stabilization during the reclamation in long term.

## Conclusions

In conclusion, our study shows that the exopolysaccharide and lipopolysaccharide‐producing bacterial community of a post‐mining agricultural reclamation chronosequence is especially favoured after disturbance events such as the initial alfalfa cultivation, and the introduction of organic fertilization and tillage during the agricultural management phase. As we hypothesized, this is accompanied by increasing exopolysaccharide concentrations in the soil. In RA6, we observed a higher concentration of exopolysaccharides compared to the initial reclamation phase, even though the absolute abundance of exopolysaccharide/lipopolysaccharide genes in RA6 did not increase, which is indicating a more efficient exopolysaccharide‐producing community. Generally, our data indicate that exopolysaccharide and lipopolysaccharide production is especially important after disturbance events, as the function is taken over by functionally redundant bacterial communities at different soil development stages.

The exopolysaccharide/lipopolysaccharide‐producing community at the initial reclamation phase was dominated by typical colonizers of oligotrophic environments, specifically nitrogen fixers (*Rhizobiaceae*, *Comamonadaceae*, *Hyphomicrobiaceae*), while bacteria common in agricultural soils such as *Sphingomonadaceae*, *Oxalobacteraceae* and *Nitrospiraceae* dominated in the agricultural management phase. *Nitrospiraceae* replaced *Sphingomonadaceae* as the most abundant exopolysaccharide producer at RA24, which underlines the advantage of bacteria involved in both exopolysaccharide/lipopolysaccharide production and important nutrient cycles over bacteria specializing on exopolysaccharide/lipopolysaccharide production solely. Our findings add to the knowledge important for an improved management of sites during ecosystem development, which could be enhanced either by stimulating polysaccharide‐producing microbes in situ, or by inoculation.

## Experimental procedures

### Study site

For this study, we chose sites of an open‐cast lignite mining area of the ‘Garzweiler’ mine (51°5′ N, 6°28′ E) located close to the city of Cologne, Germany. The mean annual temperature in the area is 9.5°C and annual precipitation is 720 mm. The typical soil type in the area is Haplic Luvisol with soil texture described as silty clay loam, and a pH of 7.4‐7.6 (Pihlap *et al.*, 2019).

During the mining process, the mining company (RWE Power AG, Essen, Germany) has been using bucket excavators to extract lignite and loess parent material. For reclamation, roughly two metres of developed Luvisol and 18 m of loess parent material (ratio of approximately 1:10) have been mixed together. The mixture has been stored in stockpiles for about three months before it has been used for the reclamation in the backside of the mine. After reclamation, the mining company managed the fields for seven years before the land was returned to the farmers.

The first three years of the reclamation process are described as the initial phase. During this time, alfalfa (*Medicago sativa*) was cultivated due to its deep rooting system and nitrogen‐fixing potential. Before the transition to the agricultural management phase, soils were tilled to a depth of 30 cm and fertilized with 30 t ha^−1^ of plant‐based organic compost in the fourth year of management. Organic fertilizer was added again in the seventh year in the form of manure, after which only mineral fertilizers have been applied. During the period of the agricultural management phase in which the fields were managed by the mining company (years four to seven), up to 1.5 t ha^−1^ of mineral fertilizers in different forms of nitrogen, phosphorus, boron, manganese, potassium and calcium were applied. A crop rotation of wheat (*Triticum aestivum *L*.*) and barley (*Hordeum vulgare *L.) was used from the fourth to the sixth year, followed by a cropping sequence of winter and summer wheat (*Triticum aestivum *L.), winter barley (*Hordeum vulgare *L.), rapeseed (*Brassica napus *L.) and maize (*Zea mays *L.), which is typical for the region.

### Soil sampling

For this study, we selected six sites in different stages of the reclamation process, simulating a chronosequence that spans 24 years of soil development (Fig. S1). Sites of the reclamation ages of 0, 1 and 3 years represent the initial phase, while the sites of the reclamation ages of 6, 12 and 24 years represent periods typical for the agricultural management phase. Throughout the manuscript, the sites are referred to as RA0, RA1, RA3, RA6, RA12 and RA24 respectively.

Bulk soil samples from the six selected sites were taken in October 2016 from the top soil layer of 0–5 cm. At the time of sampling, the site representing the reclamation age of 0 years was not yet planted, and sites of the reclamation ages of 1 and 3 years were cultivated with alfalfa. At sites representing the agricultural management phase, crops were harvested prior to sampling – wheat in RA6 and RA24, and maize in RA12. In fields that were cultivated at the time of sampling, we took extra caution to sample only bulk soil that was distant from plant roots to exclude the possibility of a direct influence of plants.

As described by Pihlap *et al. *(2019), each field (sized between 2 and 35 ha) was divided into three plots of approximately 200 m^2^. Plots were treated as biological replicates. From each plot, four soil samples were collected, homogenized and pooled. The pooled samples were shock‐frozen on dry ice and stored at −80°C until further analyses.

### Extracellular polymeric substances (EPS) extraction

To compare the quantity and quality of extracellular polymeric substances (EPS) between soils in different developmental stages of the reclamation chronosequence, extraction was performed from 2.5 g soil dry weight according to Redmile‐Gordon *et al. *(2014). The extraction technique is based on a protocol using cation exchange resin (Frølund *et al.*, 1996). This procedure was tested and confirmed to be the most selective method with the lowest cell‐damaging effects (Redmile‐Gordon *et al.*, 2014; Redmile‐Gordon *et al.*, 2015). Exopolysaccharide and protein contents of the extracts were determined as described by DuBois *et al. *(1956) and Redmile‐Gordon *et al. *(2013) using the Lowry assay.

### DNA extraction

DNA was extracted from approximately 300 mg of soil from each sample using the NucleoSpin Soil extraction kit (Macherey‐Nagel, Düren, Germany) according to the manufacturer’s protocol. Negative extraction controls were performed in each extraction run. For metagenome library preparation, DNA extraction was performed three times per sample and the extracts were pooled to ensure enough DNA material for direct sequencing even from the low biomass samples.

DNA quality was assessed photometrically by A260nm/A280nm and A260nm/A230nm measurements using a NanoDrop 1000 Spectrophotometer (PeqLab, Germany). DNA concentrations were quantified with the Quant‐iT PicoGreen dsDNA Assay Kit (ThermoFisher Scientific, Waltham, MA, USA). Extracted DNA was stored at −20°C until further processing.

### Quantitative PCR

The number of bacterial 16S genes per gram of dry weight soil was estimated by means of quantitative PCR. Quantifications were carried out in 25 µl reactions containing 12.5 µl of PowerSYBR Green Master Mix (Applied Biosystems, Germany), 0.5 µl of 3% bovine serum albumin, 0.5 µl of each primer (FP 16S 5’ – GGT AGT CYA YGC MST AAA CG – 3’, and RP 16S 5’ – GACARCCATGCASCACCTG – 3’) (Bach *et al.*, 2002) diluted to 10 µM and 2 µl sample DNA. A 7300 Real‐Time PCR System (Applied Biosystems, Germany) thermal cycler was programmed as follows: initial denaturation at 95 °C for 10 min, 40 cycles of 95°C, 58°C and 72°C and 45 s per each temperature step.

To eliminate the possible effect of PCR inhibitors, a test of series of dilutions was performed. A dilution of 1:20 was chosen for all samples, as it showed no indication of inhibition (data not shown). A serial dilution of a plasmid containing cloned 16S rRNA gene from *Pseudomonas putida* was used as standard (Gschwendtner *et al.*, 2016) and was analysed in triplicates. Several negative controls were included in the PCR run. Amplification efficiency was calculated with the equation E = [10^(−1/slope)^ – 1] and resulted in the efficiency of 92%. The analysis of the melting curve and an agarose gel confirmed the specificity of amplicons after each PCR run and showed no primer dimer formation.

### Metagenome library preparation and DNA sequencing

One hundred nanograms of DNA per sample was sheared by an E220 Focused‐ultrasonicator (Covaris, Woburn, MA, USA) according to manufacturer´s guideline for the target size of 500 bp. Conditions used were as follows: peak incident power – 175 W, duty factor – 5%, cycles per burst – 200, treatment time – 45 s, temperature – 7 °C, water level – 6, and intensifier – yes. Libraries were prepared with the NEBNext Ultra II DNA Library Prep Kit for Illumina and NEBNext Multiplex Oligos for Illumina (New England Biolabs, UK) as barcodes. The NEBNext Adaptor was diluted 10‐fold according to the manufacturer´s protocol for DNA input of ≤ 100 ng. Adapter ligated DNA was amplified in 8 PCR cycles, and PCR products were purified two times with AMPure XP beads (1:0.6 DNA to bead ratio). Library size was estimated using the Standard Sensitivity Kit for the Fragment Analyser (Advanced Analytical Technologies Inc., Ames, IA, USA). Libraries were diluted to 4 nM per sample, pooled equimolar and spiked with 1% PhiX as sequencing control. Sequencing was performed on a MiSeq instrument (Illumina Inc., San Diego, CA, USA) using the Miseq Reagent Kit v3 for 600 cycles.

### Data filtering, taxonomic and functional analysis

Raw sequences were processed as described by Vestergaard *et al. *(2017). Specifically, adapter removal and read trimming was done using AdapterRemoval (Schubert *et al.*, 2016) set to minimum Phred quality of 15, and a minimum read length of 50. PhiX contaminations were removed with DeconSeq (Schmieder and Edwards, 2011). Filtered reads were blasted against the NCBI non‐redundant protein sequences database (January 2017) for taxonomic classification. Blasting was performed with Kaiju (Menzel *et al.*, 2016) in Greedy mode with 5 allowed mismatches. Additionally, reads containing the V4 region of the 16S rRNA genes were extracted and annotated to the SILVA database (release 119) using the RiboTagger program (Xie *et al.*, 2016) for taxonomic profiling.

The average coverage of all metagenomes was estimated with Nonpareil, and Nonpareil diversity index was calculated as a proxy for describing the complexity of microbial communities (Rodriguez and Konstantinidis, 2014). We annotated 14 specific genes encoding for enzymes and proteins involved in the production of exopolysaccharides and lipopolysaccharides (Table S1) using the hidden Markov model (HMM) searches and blasts against sequences downloaded from the online Kyoto Encyclopedia of Genes and Genomes (KEGG) Orthology database (October 2016) as described by Cania *et al. *(2019). Briefly, HMMs were downloaded from the TIGRFAM (version 15) (Haft *et al.*, 2013) and Pfam (version 30) (Finn *et al.*, 2016) databases. Open‐reading frames (ORFs) prediction was performed with FragGeneScan (version 1.19) (Rho *et al.*, 2010); ORFs were scanned with HMMER (version 3) (hmmer.org). Matching reads with E‐values of less than 10^−5^ were mapped to KEGG Orthology (KO) numbers. Reads for which the top 25 blast results were consistent were assigned with a KO number. Blasting was performed with Diamond (version 0.8.38) (Buchfink *et al.*, 2014).

To estimate total abundances of genes analysed by the described metagenomic approach, we compared their mean relative abundance with mean absolute bacterial abundances measured by qPCR. Due to the variation of the 16S rRNA gene copy numbers obtained in each sample, we first calculated the ratios of 16S rRNA gene copy number in each reclamation age to RA24, where we found the highest 16S rRNA gene abundance. This was done to eliminate the qPCR bias and obtain a ‘factor’ without a unit. These factors (ratios) were then multiplied by the relative abundance of the analysed exopolysaccharide and lipopolysaccharide genes.

### Statistical analysis

Statistical analysis and data visualization were performed with the R v3.5.1 statistical software (R Core Team, 2018). Due to the variation of total read counts between samples, all statistical analyses of sequencing results were based on relative read abundances. Differences in exopolysaccharide and protein concentrations, bacterial abundances and gene relative abundances between reclamation ages were tested with a robust one‐way ANOVA with trimmed means using the *t1way* function, and a post hoc *lincon* function from the WRS2 package, which contains robust statistical methods that do not rely on parametric assumptions (Mair and Wilcox, 2018). All differences were considered significant when the *P* value was <0.05 after Benjamini–Hochberg correction (Benjamini and Hochberg, 1995).

## Conflict of interests

The authors declare that they have no competing interests.

## Supporting information


**Figure S1** Section of the Garzweiler open‐pit mine (crossed out) and studied reclamation areas, modified from Pihlap *et al.* (2019).
**Figure S** **2** Protein fraction of the extracellular substance calculated as equivalent of bovine serum albumin used as the standard, per gram of dry soil. Bars represent the means of triplicates with a standard deviation.
**Figure S** **3** Sequencing summary showing the average number of raw and filtered reads of all metagenomes, the number of filtered reads per sample, and the average read length per sample.
**Figure S** **4** Distribution of reads assigned to genera within the most abundant families. Shown are mean abundances relativized to the number of bacterial reads.
**Figure S** **5** Distribution of the most abundant bacterial families based on the 16S rRNA gene (V4 region), as assigned by Ribotagger and SILVA database.
**Figure S** **6** Absolute gene abundance estimation.
**Table S** **1** Analysed genes related to exo‐ and lipopolysaccharide production, the proteins they code for, and their respective KO numbers and HMM IDs.
**Table S2** Nonpareil coverage estimation and diversity index per replicate.

## Data Availability

The raw sequencing data is deposited to the Sequence Read Archive (SRA) and is available under the BioSample accession numbers SAMN12404105‐22. These accession numbers are linked to the BioProject PRJNA557612.
