# Supplementary material for: Shifts in reclamation management strategies shape the role of exopolysaccharide and lipopolysaccharide‐producing bacteria during soil formation
Source: Microb Biotechnol. 2020 Jan 9;13(2):584–98. doi: 10.1111/1751-7915.13532 (PMC7017822; doi:10.1111/1751-7915.13532)
Supplement: Supplementary file 1 — Figure S1 Section of the Garzweiler open‐pit mine (crossed out) and studied reclamation areas, modified from Pihlap et al. (2019). Figure S 2 Protein fraction of the extracellular substance calculated as equivalent of bovine serum albumin used as the standard, per gram of dry soil. Bars represent the means of triplicates with a standard deviation. Figure S 3 Sequencing summary showing the average number of raw and filtered reads of all metagenomes, the number of filtered reads per sample, and the average read length per sample. Figure S 4 Distribution of reads assigned to genera within the most abundant families. Shown are mean abundances relativized to the number of bacterial reads. Figure S 5 Distribution of the most abundant bacterial families based on the 16S rRNA gene (V4 region), as assigned by Ribotagger and SILVA database. Figure S 6 Absolute gene abundance estimation. Table S 1 Analysed genes related to exo‐ and lipopolysaccharide production, the proteins they code for, and their respective KO numbers and HMM IDs. Table S2 Nonpareil coverage estimation and diversity index per replicate. [file MBT2-13-584-s001.docx]

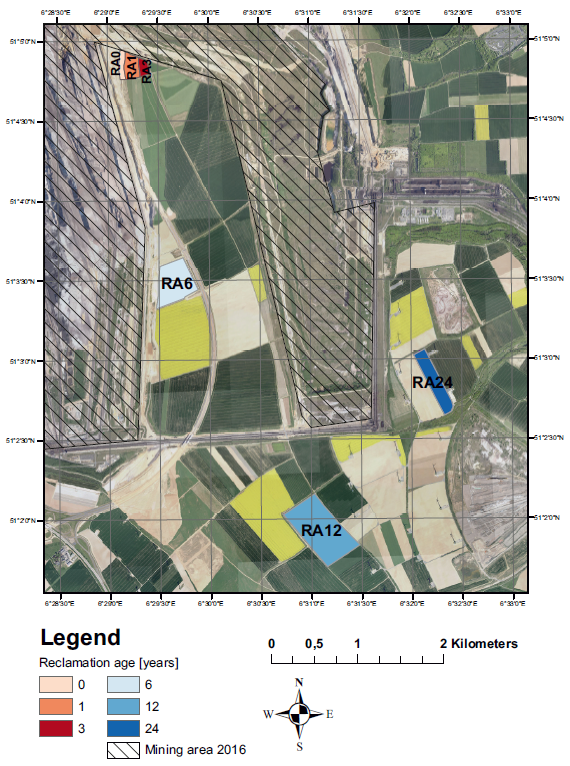


**Supplement figure 1.** Section of the Garzweiler open-pit mine (crossed out) and studied reclamation areas, modified from Pihlap *et al.* (2019).


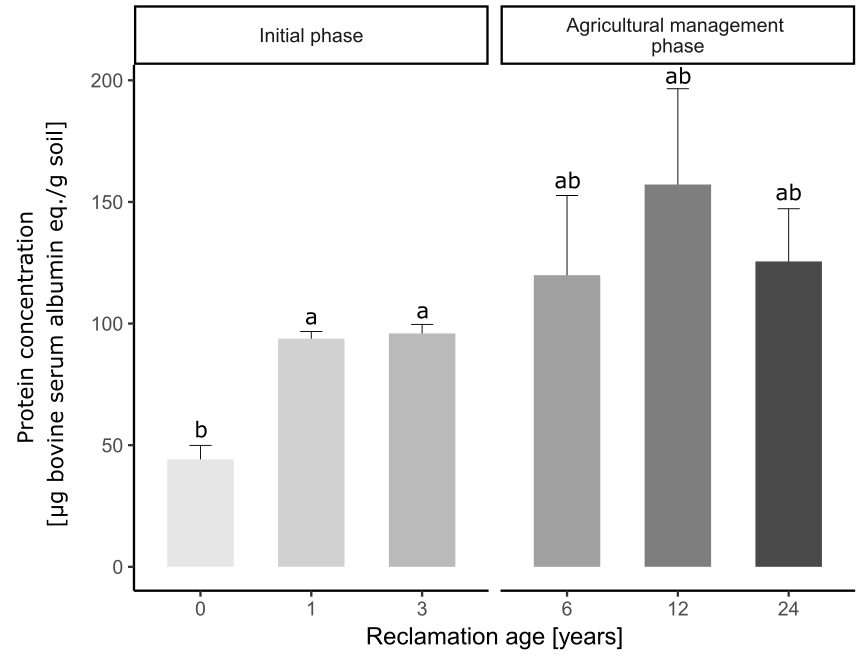


**Supplement figure 2.** Protein fraction of the extracellular substances calculated as equivalent of bovine serum albumin used as the standard, per gram of dry soil. Bars represent the means of triplicates with a standard deviation. Letters above bars represent pairwise comparisons adjusted with the Benjamini-Hochberg correction showing the significant differences between reclamation ages after a robust ANOVA test with trimmed means (p < 0.005).


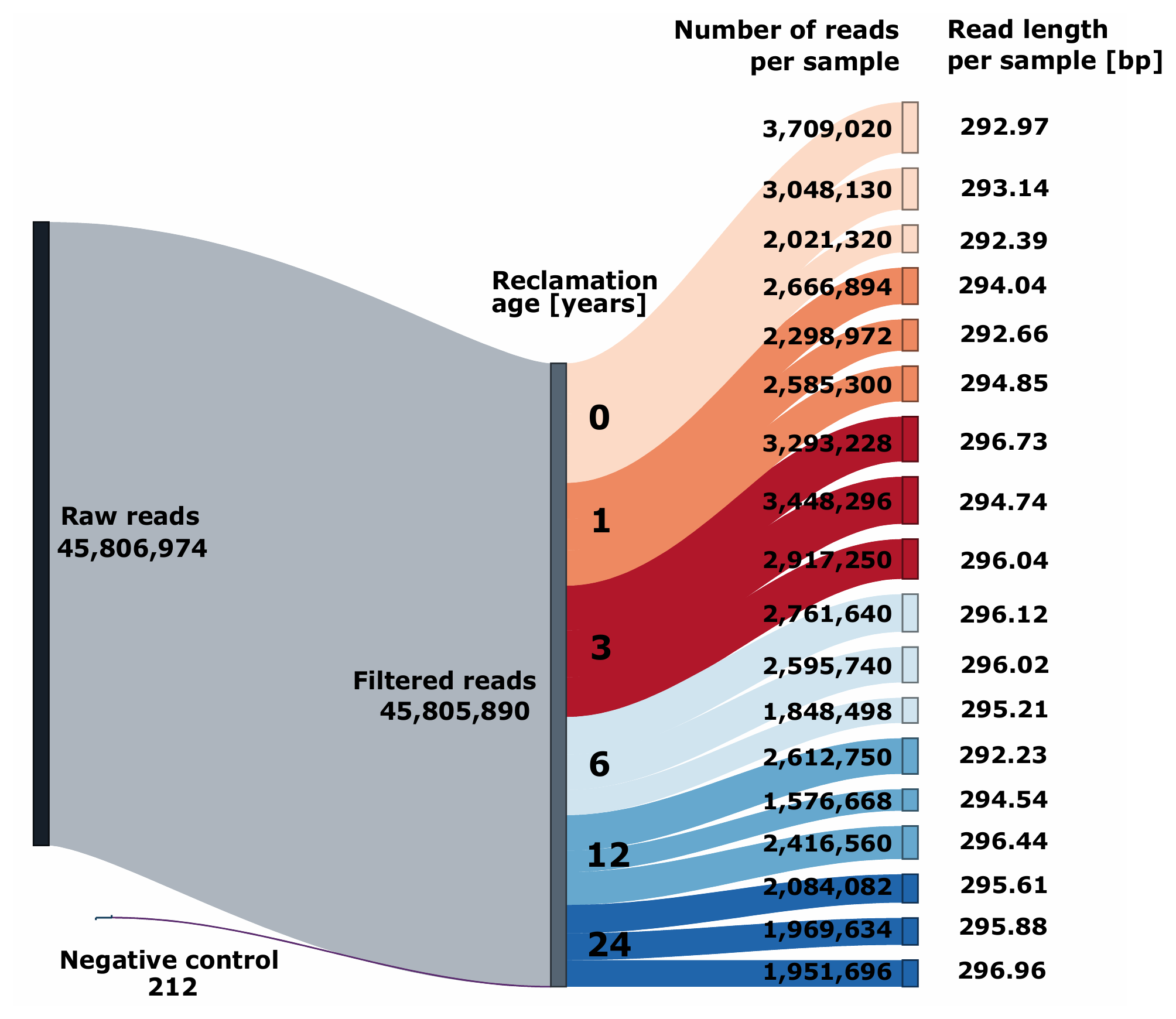


**Supplement figure 3.** Sequencing summary showing the average number of raw and filtered reads of all metagenomes, the number of filtered reads per sample, and the average read length per sample.


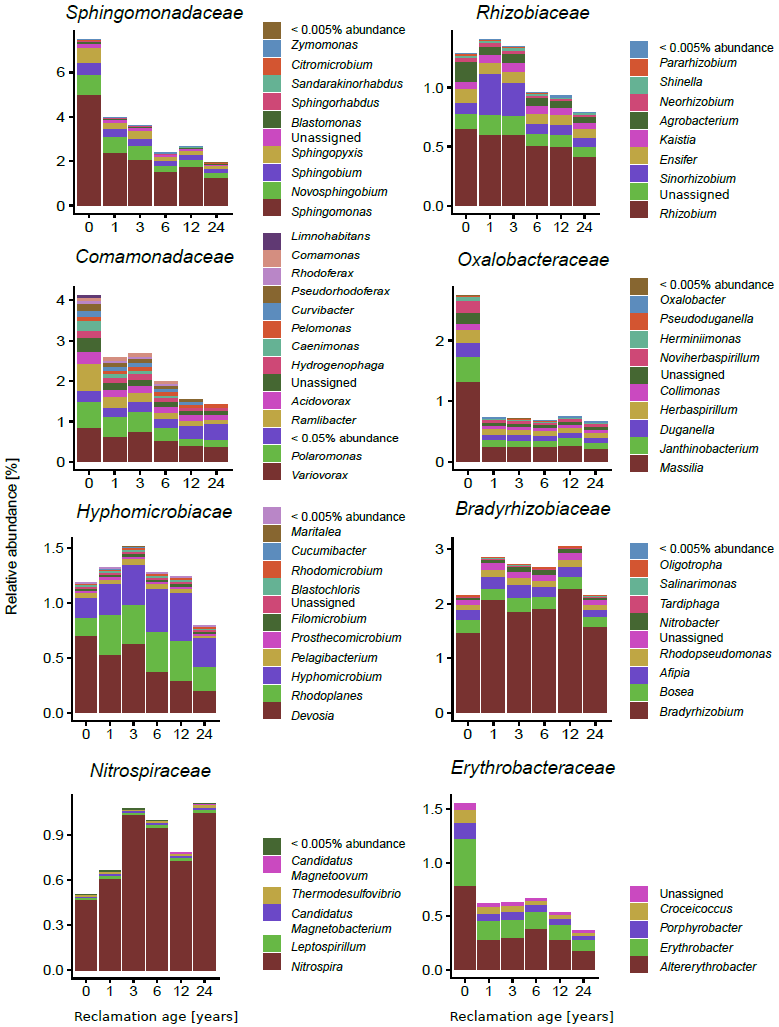


**Supplement figure 4.** Distribution of reads assigned to genera within the most abundant families. Shown are mean abundances relativized to the number of bacterial reads.


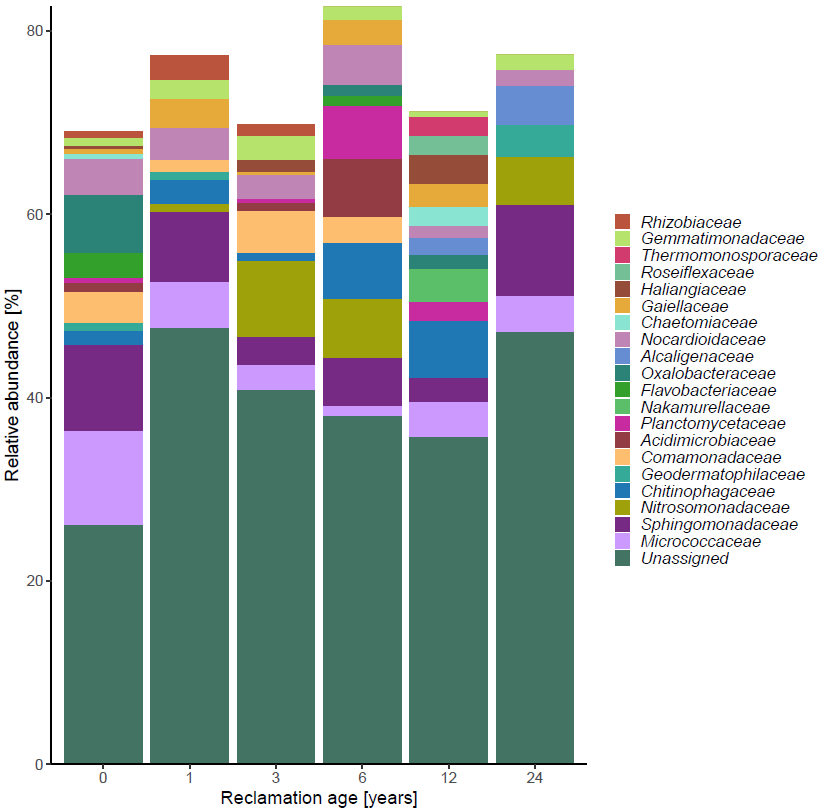


**Supplement figure 5.** Distribution of the most abundant bacterial families based on the 16S rRNA gene (V4 region), as assigned by Ribotagger and SILVA database. Shown are mean abundances relativized to all reads assigned to the family level.


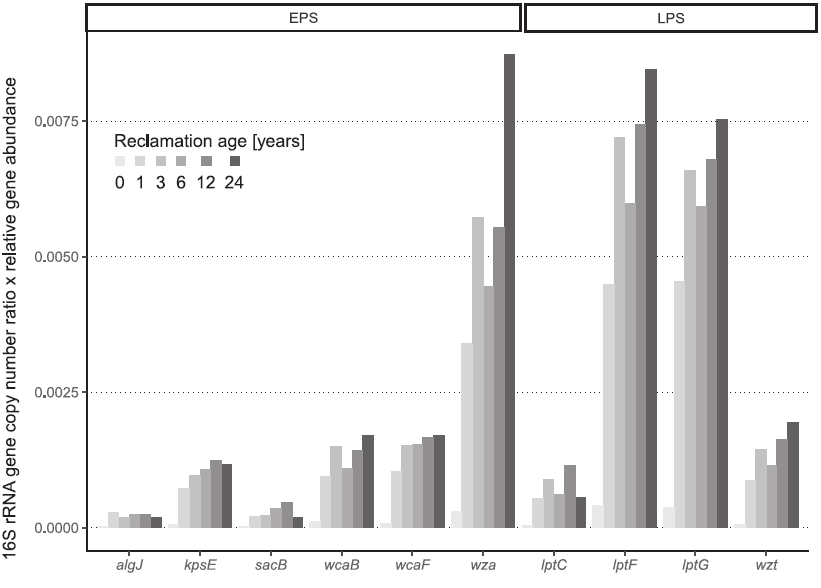


**Supplement figure 6.** Absolute gene abundance estimation. The mean value of 16S rRNA gene copy number in each reclamation age was divided by the highest mean value (found in RA24). Resulting ratios were multiplied with the mean relative abundance of each gene analysed from the metagenome to obtain a rough estimation of the absolute gene abundance in each reclamation age compared to RA24.

| **Gene** | **Protein** | **KO number** | **HMM ID** |
| --- | --- | --- | --- |
| *wza* | polysaccharide export outer membrane protein - Wza | K01991 | PF02563 |
| *algE* | alginate export outer membrane protein - AlgE | K16081 | PF13372 |
| *algJ* | alginate biosynthesis acetyltransferase - AlgJ | K19295 | PF16822 |
| *wcaB* | colanic acid biosynthesis acetyltransferase - WcaB | K03819 | TIGR04016 |
| *wcaF* | colanic acid biosynthesis acetyltransferase - WcaF | K03818 | TIGR04008 |
| *wcaK/amsJ* | colanic acid/amylovoran biosynthesis pyruvyl transferase - WcaK/AmsJ | K16710 | TIGR04006 |
| *kpsE* | capsular polysaccharide export system permease - KpsE | K10107 | TIGR01010 |
| *epsG* | exopolysaccharide biosynthesis transmembrane protein - EpsG | K19419 | PF14897 |
| *epsA* | exopolysaccharide biosynthesis tyrosine kinase modulator - EpsA | K19420 | TIGR01006 |
| *sacB* | levansucrase - SacB | K00692 | PF02435 |
| *wzt* | lipopolysaccharide transport system ATP-binding protein - Wzt | K09691 | PF14524 |
| *lptF* | LptBFGC lipopolysaccharide export complex permease - LptF | K07091 | TIGR04407 |
| *lptG* | LptBFGC lipopolysaccharide export complex permease - LptG | K11720 | TIGR04408, PF03739 |
| *lptC* | LptBFGC lipopolysaccharide export complex inner membrane protein - LptC | K11719 | TIGR04409, PF06835 |

**Supplement table 1.** Analysed genes related to exo- and lipopolysaccharide production, the proteins they code for, and their respective KO numbers and HMM IDs.

|  | **Reclamation age [years]** | **Coverage [%]** | **Diversity index** |
| --- | --- | --- | --- |
| Initial phase | 0 | 3.3^a^ | 22.9^a^ |
|  | 0 | 5^a^ | 22.51^a^ |
|  | 0 | 4^a^ | 22.81^a^ |
|  | 1 | 2.3^ab^ | 25.35^b^ |
|  | 1 | 3^ab^ | 27^b^ |
|  | 1 | 4.9^ab^ | 25.86^b^ |
|  | 3 | 1.7^ab^ | 24.15^a^ |
|  | 3 | 2.9^ab^ | 22.79^a^ |
|  | 3 | 3^ab^ | 24.42^a^ |
| Agricultural management phase | 6 | 1.5^b^ | 22.59^a^ |
|  | 6 | 1.5^b^ | 22.54^a^ |
|  | 6 | 1.6^b^ | 22.35^a^ |
|  | 12 | 0.08^b^ | 22.49^a^ |
|  | 12 | 1.4^b^ | 22.11^a^ |
|  | 12 | 1.2^b^ | 22.72^a^ |
|  | 24 | 1^b^ | 22.6^a^ |
|  | 24 | 2.5^b^ | 22.27^a^ |
|  | 24 | 2^b^ | 22.25^a^ |
|  |  |  |  |

**Supplement table 2.** Nonpareil coverage estimation and diversity index per replicate. Letters represent pairwise comparisons showing the significant differences between reclamation ages after a robust ANOVA test with trimmed means (p = 0.049, p = 0.014 for coverage and diversity, respectively).
